# Physiologically Based Pharmacokinetic Modeling to Predict Lamotrigine Exposure in Special Populations to Facilitate Therapeutic Drug Monitoring and Guide Dosing Regimens

**DOI:** 10.3390/ph18050637

**Published:** 2025-04-27

**Authors:** Ji-Cheng Li, Chen-Fang Miao, Yun Lei, Ai-Lin Liu

**Affiliations:** Higher Educational Key Laboratory for Nano Biomedical Technology of Fujian Province, Department of Pharmaceutical Analysis, The School of Pharmacy, Fujian Medical University, Fuzhou 350122, China

**Keywords:** lamotrigine, physiologically based pharmacokinetic model, special population, therapeutic drug monitoring, drug exposure

## Abstract

**Background**: Lamotrigine plays a crucial role in the treatment of epilepsy and bipolar disorder in adults and children. However, its pharmacokinetic (PK) behavior in first or long-term treatment in pediatric patients and the changes in drug exposure in patients with renal impairment are not well characterized. The purpose of the research was to build a robust physiologically based pharmacokinetic (PBPK) model of lamotrigine for the prediction of drug exposure in diverse populations to facilitate therapeutic drug monitoring (TDM) and guide dosing regimens. **Methods**: The physicochemical parameter values of lamotrigine were integrated to establish and validate the model in an adult population in PK-sim. This adult PBPK model can be extrapolated to children and patients with renal impairment to predict PK changes. **Results**: Most of the observed data were within the 5th and 95th percentile intervals of the variability around the predicted plasma concentrations. The model predicted pharmacokinetic thresholds and exposure values for clinically safe and effective doses recommended by the FDA for initial and long-term treatment of epilepsy in adults and children aged 2–12 years. Notably, patients with severe renal impairment and end-stage renal disease experienced an average increase in the area under the curve of 1.51 folds and 1.62 folds, respectively. This scenario necessitates further lamotrigine dose adjustments. **Conclusions**: The developed lamotrigine PBPK model offers a strategy for assisting clinicians in TDM and dose adjustment for special populations, thereby offering a reference (PK parameters, as well as peak and valley concentrations to reach a steady state) for a safer administration regimen in clinical treatment.

## 1. Introduction

Epilepsy is a paroxysmal chronic nervous system disease caused by highly synchronized and self-limited abnormal discharge of brain neurons [1]. It ranks as the second most common chronic neurological disease and is characterized by recurrent, episodic, transient, and stereotypical dysfunction within the central nervous system [2,3,4]. In addition, epileptic seizures present complex and varied clinical manifestations, including muscle spasms, limb tetanic convulsions, and loss of consciousness [5,6]. According to the Global Report on Epilepsy published by the World Health Organization (WHO) in 2019, it is estimated that there are nearly 50 million epilepsy patients worldwide, with an annual increase of about 400,000 new cases [7]. Epilepsy significantly affects the overall quality of life of patients and imposes a substantial economic burden on both families and society.

Lamotrigine, a phenyltriazine compound, has been approved as an oral tablet by the US Food and Drug Administration (FDA) [8]. Due to its unique mechanism of action and the rapid onset of its effects, it is recommended as a therapeutic intervention for individuals diagnosed with epilepsy and bipolar disorder [9]. Several prospective, randomized, controlled clinical studies have demonstrated the favorable efficacy, tolerability, and safety of lamotrigine in the management of epilepsy in patients [10,11]. Notwithstanding its therapeutic benefits, lamotrigine may elicit certain unfavorable responses, including dizziness, somnolence, fatigue, and headache. Clinical monitoring of lamotrigine plasma concentrations is needed to prevent adverse events during the treatment of epilepsy patients [12]. In addition to therapeutic drug monitoring (TDM), the characteristics of individual patients should be comprehensively evaluated, especially in special populations [13].

The intricate and diverse physiological changes in special populations, such as children, individuals with renal disorders, pregnant women, and patients under the influence of enzyme-inhibiting or -inducing drugs, significantly influence the in vivo disposition and exposure of drugs. Although the effectiveness and safety of lamotrigine have been extensively studied in adults, the clinical data on lamotrigine, especially regarding its PK behavior, in special populations are relatively limited, such that these vulnerable populations may be exposed to potential dosing risks, including suboptimal therapeutic outcomes and adverse drug reactions. It is neither ideal nor feasible to conduct extensive in vivo PK studies in special populations due to the ethical considerations and technical constraints. Hence, it is imperative to devise a novel approach for comprehending the PK behavior of drugs in specific populations and reasonably predicting drug exposure.

Recently, physiologically based pharmacokinetic (PBPK) modeling and simulations have become increasingly prevalent for the estimation of alterations in drug PK behavior and new drug development guidance [14]. The concept of the PBPK model was proposed by Teorell, a Swedish physiologist and biophysicist, in 1937 [15]. This model, based on anatomy, physiology, and biochemistry, integrates physicochemical properties of drugs with relevant clinical parameters to simulate drug disposal within the body and further investigate processes of drug absorption, distribution, metabolism, and excretion. A PBPK model that accounts for the physiological alterations in special populations can serve as a dependable tool for assessing PK attributes in diverse populations, even those with medical conditions, and deriving appropriate dosage recommendations [16,17]. Regulatory bodies such as the FDA, the European Medicines Agency, and the International Council for Harmonization have suggested the utilization of modeling and simulation as a means of directing drug development and determining appropriate dosages for special populations [18].

Lamotrigine is utilized as a long-term therapeutic agent for epilepsy; however, it carries risks, particularly if the initial dosage is excessively high or if the dosage is increased too rapidly. Gradual titration and TDM can mitigate the risk of toxicity and adverse effects. Considering the intrinsic variability among patients, clinical practice necessitates accounting for individual patient characteristics in drug metabolism, particularly within special populations. Monitoring blood concentrations serves as a crucial reference point for these groups. The PBPK model emerges as a strategic tool that aids in assessing the scientific rigor and comprehensiveness of a drug prior to treatment while simultaneously offering clinicians valuable information to support the rational use of the drug.

**Figure 1:**
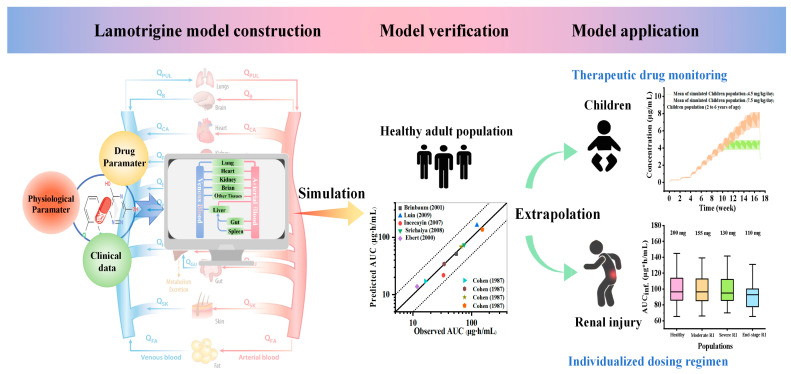
Schematic diagram of physiologically based pharmacokinetic model of lamotrigine in special populations to assist therapeutic drug monitoring and guide dosing regimens [22,23,24,25,26,27].

To date, research has been conducted on the development of lamotrigine models in specific populations, predominantly focusing on pediatric subjects. These studies primarily investigated the impact of pertinent gastrointestinal physiological parameters, drug formulation solubility, and breastfeeding practices on lamotrigine pharmacokinetics [19,20,21]. However, there is a notable scarcity of data concerning lamotrigine concentrations during the initial treatment phase, throughout prolonged administration, and under steady-state conditions. Furthermore, there is limited knowledge concerning the change in lamotrigine exposure in renally impaired populations. The present study established a lamotrigine PBPK model and subsequently verified its reliability. This model extrapolation was implemented for patients taking lamotrigine medication for the first time and special populations, such as the renally impaired population, with the aim of estimating the drug PK behavior and plasma exposure and thresholds for treatment with and the toxicity of lamotrigine. The established model will serve as a valuable tool for supplementing the limited clinical PK data in special patient populations and can be applied to facilitate TDM and optimize dosing regimens for epilepsy treatment by simulation studies on lamotrigine concentration changes (Figure 1).

## 2. Results

### 2.1. Establishment and Evaluation of Lamotrigine PBPK Model in Healthy Adults

**Figure 2:**
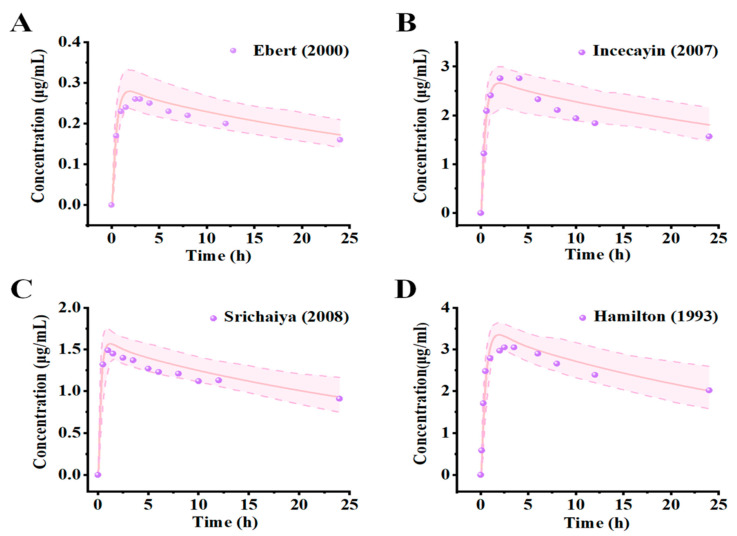
Simulations of pharmacokinetics of lamotrigine at a series of doses in healthy humans after a single oral administration. Prediction of plasma concentration–time profiles in healthy humans with dosages of 25 ((**A**), [26]), 100 ((**B**), [24]), 200 ((**C**), [25]), 300 ((**D**), [28]) compared with the observed reference data. The observed data are from published clinical studies and are shown as solid purple circles. The pink lines represent the simulated mean plasma concentration–time profiles. The shaded areas of light pink represent the 5th and 95th confidence intervals of the simulated mean concentrations.

**Figure 3:**
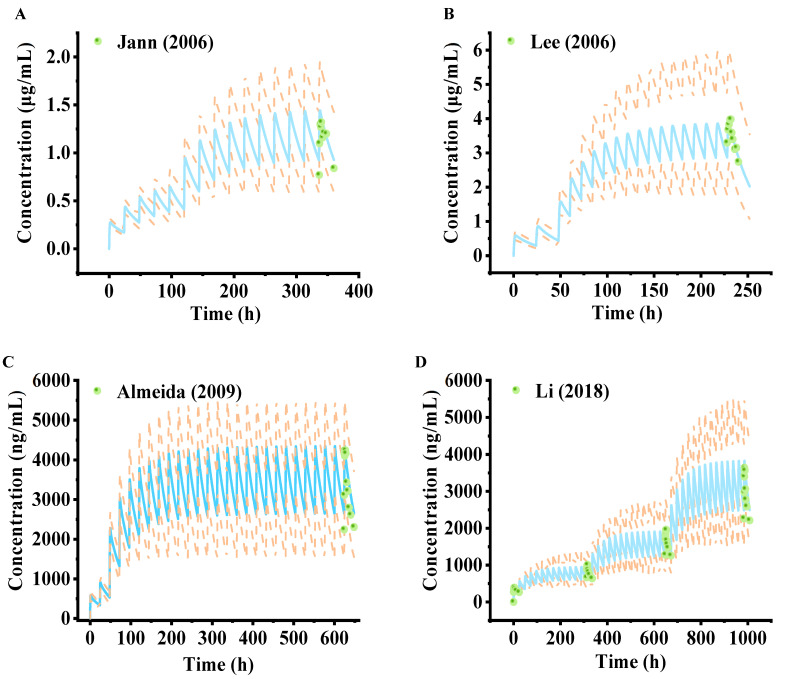
The predictions of lamotrigine mean plasma concentration–time profiles in healthy humans after multiple oral administrations compared with the observed reference data: ((**A**–**D**), [29,30,31,32]). The observed data are from published clinical studies and indicated as solid green circles. The light-blue lines represent the simulated mean plasma concentration–time profiles. The areas between the two orange dotted lines represent the 5th and 95th confidence intervals of the simulated mean concentrations.

**Figure 4:**
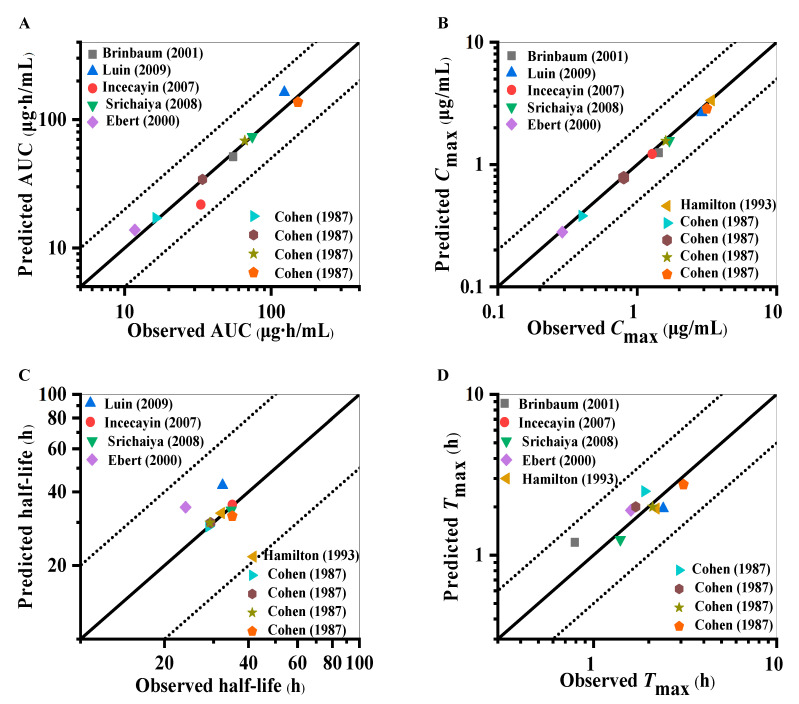
The goodness-of-fit plots for the PBPK model of healthy adults with single dosages obtained by comparison of the observed pharmacokinetic parameters with the predicted parameters [22,23,24,25,26,27,28]. (**A**) AUC. (**B**) *C*_max_. (**C**) Half-life. (**D**) *T*_max_. Each point represents the observed value versus the predicted value. The solid black lines indicate the lines of identity; the black dotted lines indicate 2-fold deviations.

**Figure 5:**
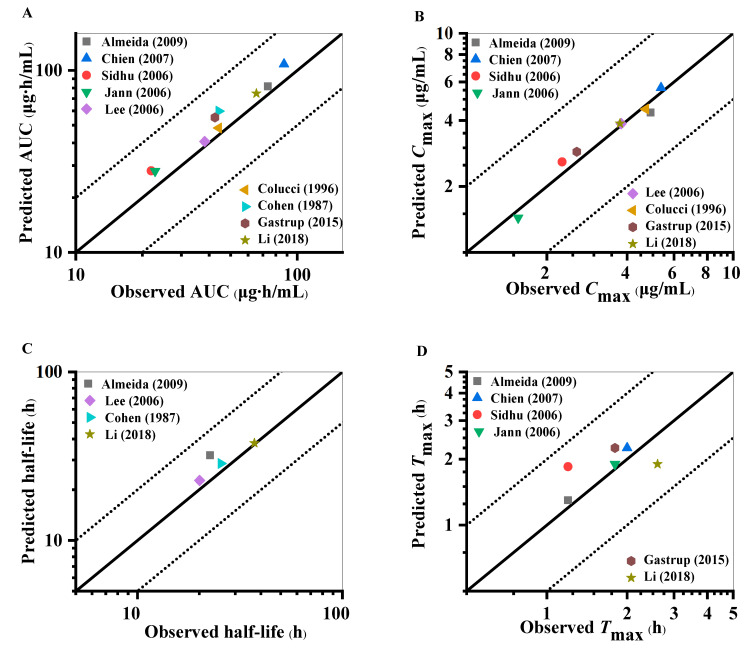
The goodness-of-fit plots for the PBPK model of healthy adults with multiple dosages obtained by comparison of the observed pharmacokinetic parameters with the predicted parameters [27,29,30,31,32,33,34,35,36]. (**A**) AUC. (**B**) *C*_max_. (**C**) Half-life. (**D**) *T*_max_. Each circle represents the observed value versus the predicted value. The solid black lines indicate the lines of identity; the black dotted lines indicate 2-fold deviations.

Visual predictive checks suggest that the predicted mean PK profile fitted well with the observed clinical values, as presented in Figure 2 and Figure S1. Most of the observed points for the predicted mean of the concentration–time curve fell within the 5th and 95th percentile intervals of the variability, substantiating the model’s predictive performance in forecasting the dispositional character of the drug within a dose range of 25 mg to 300 mg. Figure 3 and Figure S2 show the model validation plots and simulated PK profiles after multi-dose oral administration of lamotrigine, thereby confirming the predictive power of this model. The goodness-of-fit plots (Figure 4 and Figure 5), derived from the comprehensive data presented in Tables S1 and S2, provide visual confirmation of the PBPK model’s robustness in accurately predicting the majority of clinical data associated with lamotrigine. Furthermore, the predicted area under the curve (AUC), *C*_max_, half-life, and *T*_max_ values fell within a range of 0.5 to 2.0 folds of the observed AUC values in the corresponding studies. This suggests that the construction and validation of the lamotrigine PBPK model in adults was successful, as generally acknowledged.

### 2.2. Sensitivity Analysis

To determine the most sensitive parameters in the model, we performed a sensitivity analysis based on a simulated oral administration of 100 mg of lamotrigine. The sensitive parameters of lamotrigine’s AUC (Figure S3A) and *C*_max_ (Figure S3B) are shown in Figure S3. For the global parameter AUC analysis, the parameter sensitivity analysis of the 179 PBPK model parameters showed that the most influential parameters included dose, kidney–body weight, kidney–plasma clearance, and so on. For the total parameter *C*_max_ analysis, the most influential parameters were dose, lipophilicity, fraction unbound, pKa, and so on. Sensitivity values greater than 1.0 indicated that the test model parameters had a significant effect on the predicted AUC and *C*_max_. Overall, sensitivity analysis showed that most modeling parameters had little effect on the AUC and *C*_max_ of lamotrigine.

### 2.3. Establishment and Assessment of the Lamotrigine PBPK Model in Pediatrics

**Figure 6:**
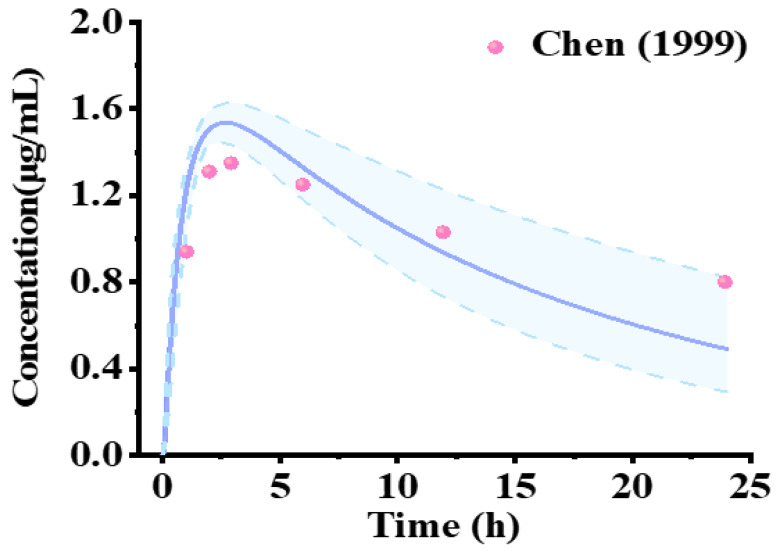
Model-predicted mean plasma concentration–time profiles for lamotrigine in children who received a single oral administration. The observed data are from a published clinical study [37] and indicated as solid red circles. The purple line represents the simulated mean plasma concentration–time profile. The area between the two light-blue dotted lines represents the 5th and 95th confidence intervals of the simulated mean concentrations.

After verifying the model in healthy volunteers, the adult PBPK model was extended to the pediatric population by integrating physiological and modality-specific ontogeny changes (Table S3). The lamotrigine PK profiles in children aged 2 to 12 years, following a single dose, were utilized to validate the pediatric PBPK model (Figure 6). The model’s validity was visually confirmed by comparing the predicted and observed PK parameters. The majority of the observed points were within the 5th and 95th percentile ranges, indicating successful extrapolation of the model to the pediatric population. The simulation exhibited satisfactory performance in forecasting the data among children aged under and over 6 years old. The fold errors for the AUC, *C*_max_, half-life, and *T*_max_ ranged from 0.5 to 2.0, indicating that the lamotrigine-based dosing regimen was sufficient to achieve similar PK behavior in the pediatric subjects (Table S4).

### 2.4. Application of the PBPK Model of Lamotrigine for Facilitating Therapeutic Drug Monitoring in Clinical Treatment

Additional exploratory simulations were performed on the established adult and pediatric models to investigate drug exposure for facilitating TDM in clinical treatment. The drug-specific characteristics were maintained unaltered. The physiological parameters and population characteristics were set according to the actual clinical conditions of diverse populations in different periods. A model strategy for lamotrigine applicable to diverse populations was used to simulate and evaluate the therapeutic PK parameters of dosing regimens recommended by the WHO and the US FDA for adults and children aged 2 years and older. The PK simulation, depicted in Figure S4, utilizing the FDA-recommended adult and child doses, facilitates the investigation of the range of blood concentrations and drug exposure for first or long-term treatment, thereby providing valuable insights for TDM in clinical practice.

### 2.5. Simulated Pharmacokinetic Profiles in Populations with Renal Impairment

**Figure 7:**
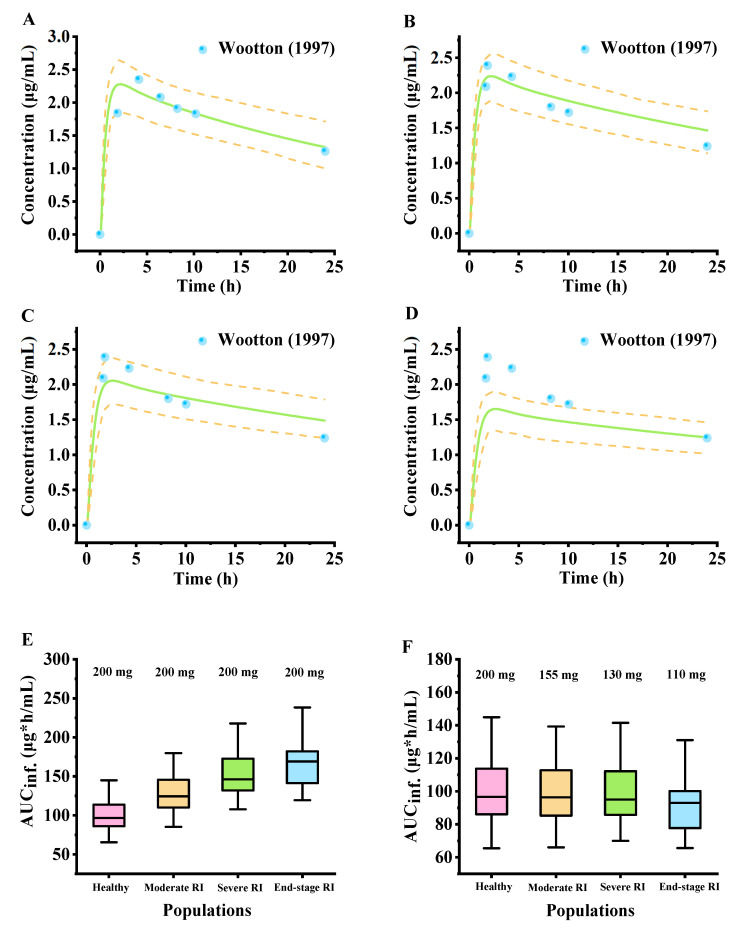
Model-predicted mean plasma concentration–time profiles in adults with different renal impairment severities following single oral administration of 200 mg of lamotrigine. (**A**) Normal renal function. (**B**) Moderate renal impairment. (**C**) Severe renal impairment. (**D**) End-stage renal disease [38]. Box–whisker analysis of exposure following single oral administration of 200 mg of lamotrigine (**E**) or adjusted dosing (**F**) for healthy individuals and patients with renal injury using the PBPK model of lamotrigine. The observed data are from published clinical studies and indicated as solid blue circles. The green lines represent the simulated mean plasma concentration–time profiles. The areas between the two yellow lines represent the 5th and 95th confidence intervals of the simulated mean concentrations.

After validating the model in healthy subjects, the primary physiological alterations associated with renal injury were integrated into a disease-specific model to illustrate the application of the model to inform the selection of doses for patients with renal impairment (Table S5). Figure 7A–D illustrate that PBPK modeling predicted the impact of varying degrees of renal dysfunction on the plasma concentration of lamotrigine subsequent to a single oral dose of 200 mg (Table S6). Box–whisker plots were employed to assess the impact of disease severity on AUC elevation in renal impairment patients. The AUC values for plasma following a single oral administration of 200 mg of lamotrigine increased with renal injury severity compared with those for healthy adults (Figure 7E). The lamotrigine doses in different renal injury groups were adjusted on the basis of AUC prediction in the healthy population to achieve comparable plasma exposure. To achieve the same treatment effect as in the healthy population, the recommended dose should be adjusted to approximately 78% for moderate renal impairment, 65% for severe renal impairment, and 55% for end-stage renal disease populations (Figure 7F). The simulations conducted by the model aimed to address the data deficiencies due to the absence of clinical PK data for patients with varying degrees of renal impairment. The findings have noteworthy implications, particularly with regard to clinical dosage regimens and adverse drug reactions in populations with renal injury.

## 3. Discussion

The present study established an oral PBPK model for lamotrigine, which was adequately evaluated and described in vivo among adults. Pharmacokinetic thresholds and exposure values were fully evaluated for clinically safe and effective doses recommended by the FDA for initial and long-term treatment of epilepsy in adults and children aged 2–12 years. Based on the adult model, PBPK models were utilized to account for pharmacokinetic profiles in populations with renal impairment and provide dosing recommendations for lamotrigine in patients suffering from varying degrees of impaired kidney function.

Through the use of clinical studies not employed in the initial model development as external validation and evaluation tools, we observed that the simulated pharmacokinetic spectra matched the empirical results (Figure 2 and Figure S1). Furthermore, the model not only predicts and corroborates the PK curve shape but also assesses key PK parameters such as the AUC, as well as drug peak time and half-life, which are applicable to other drug disposition scenario evaluations. The results show that the PK parameters simulated by the lamotrigine PBPK model of healthy adults meet the 2-fold error threshold standard (Figure 4). Nevertheless, the accuracy of multi-dose simulation predictions can be influenced by several factors, including model performance, data extraction from observations, physiological alterations during administration, limited sampling points, imprecise sampling times, and other variables. Despite these, the multi-dose simulation validation results demonstrated that the critical parameters influencing lamotrigine’s PK behavior and its disposition process are comprehensively captured (Figure 3, Figure 5, and Figure S2). Typically, the administration of lamotrigine involves a gradual escalation of dosage, and multi-dose simulation can better demonstrate the steady-state process of plasma concentration from low to high doses. This method is expected to assist clinicians in personalized dose prediction and TDM of long-term epilepsy treatment. The results of the software for the actual clinical applications may be biased with respect to the actual monitoring values due to the differences in physiological function between individual patients, multi-drug therapies, and complex underlying diseases.

Obtaining drug exposure data for the pediatric population is difficult, which makes determining appropriate dosing regimens for pediatric patients a challenge in pediatric drug development [39,40]. The pediatric PBPK model demonstrated a reasonable ability to replicate the PK profiles of lamotrigine in pediatric subjects (Figure 6). In clinical practice, pediatric dosing is weight-adjusted, typically resulting in lower total drug doses than in adults but often yielding comparable or even higher plasma concentrations, as evidenced by model studies (Figure S4). This may be attributed to the reduced plasma volume and distribution space in children. The relationship between increased gastric emptying time and the resultant lower predicted *C*_max_ and increased *T*_max_ values warrants further investigation, particularly in children aged 2–6 years. It remains unclear whether the observed alterations in absorption patterns in this age group are attributable to slower gastric emptying or other contributing factors. Notably, oral drug absorption is dependent on important physiological variables, such as pH-dependent fusion, gastric emptying time, and gastrointestinal motility, all of which show age-dependent progression [41,42]. Moreover, the exposure–safety relationships for pharmacological agents can vary, with pediatric patients potentially demonstrating heightened sensitivity to adverse effects compared to adults. Consequently, blood concentrations and drug exposures in epilepsy patients need to be assessed to inform the design of safe and effective dosing regimens for both initial treatment and long-term therapeutic administration.

Lamotrigine is typically administered on a long-term basis for the management of epilepsy [43]. In clinical practice, the dosing regimen is incrementally adjusted based on therapeutic response, such as achieving optimal seizure control, and subsequently maintained at a stable dose. For patients newly diagnosed with epilepsy, it is imperative to incrementally increase the dosage with caution in order to minimize the risk of surpassing the steady-state concentration range at the maximum recommended dose [44]. Consequently, it is essential to elucidate the PK properties of the drug following multiple dosing regimens.

To avoid a lack of publicly available data, our model incorporates the recommended regimen for adults and children listed on the FDA label to assess steady-state drug concentrations and exposure over the dosing range as a reference for the initial dosing regimen (Figure S4). The model results forecast the spectra of therapeutic concentrations and establish safe upper limits for the initial administration to homeostasis in both adults and children across various age groups, which align with the TDM guidelines established by the International League Against Epilepsy (ILAE), as well as the recommended reference concentrations for lamotrigine in Arbeitsgemeinschaft für Neuropsychopharmakologie und Pharmakopsychiatrie (AGNP) for adult epilepsy treatment, which range from 3 mg/L to 15 mg/L. These findings also address the previously absent reference range for pediatric populations. This information can assist clinicians in determining threshold concentrations corresponding to different dosages, thereby facilitating the rationalization of drug titration for both new and long-term patients. Furthermore, based on the patient’s clinical manifestations, clinicians can adjust the medication type, dosage, and frequency to enhance the management of epilepsy, thereby preventing seizures and minimizing the patient’s exposure to unnecessary adverse events. Due to the large variation between individuals, it cannot completely resolve and replace the importance of TDM. Previous clinical trials suggest that caution is warranted when titrating doses beyond the recommended upper limit, necessitating careful monitoring for any adverse effects [45,46]. The toxicity of lamotrigine progressively escalates with rising serum concentrations, while the occurrence of adverse events markedly intensifies at concentrations exceeding 13–14 mg/L. The evidence supporting a correlation between lamotrigine exposure and its efficacy, such as seizure control, is limited. Furthermore, the absence of clinical studies on higher doses leaves the risk of adverse effects unclear. The lamotrigine model, when used in conjunction with TDM, serves as an invaluable tool in patient management. It facilitates the implementation of informed simulations of pre-treatment during the optimization of drug regimens. Clinicians make an assessment according to the body weight and other available physiological characteristics of individual child patients, enabling personalized prediction of the blood drug concentration of individual patients after administration through the software to assist TDM. However, the differences between unavailable individual physiological parameters and the built-in parameters of the software may affect drug absorption and metabolism. Meanwhile, the complexity of the child’s individual development process will also affect the software’s prediction and monitoring of drugs. This approach aids in estimating drug concentration ranges for the design of dosing regimens, with the objectives of optimizing clinical outcomes, minimizing adverse effects, and supporting clinicians in making individualized treatment decisions.

Accumulating evidence in forecasting PK behavior in individuals with chronic kidney disease suggests the potential of PBPK models as a substitute for clinical trials in adjusting drug administration for renal elimination [47]. Lamotrigine is primarily excreted in the urine, so any changes in renal function will affect the PK behavior of lamotrigine. In this study, adjustments were made to the physiological parameters of the PBPK model in response to the deterioration in renal function, while the physicochemical parameters remained unchanged. The findings indicate that the decline in kidney function had a restricted impact on *C*_max_ but a substantial impact on AUC, resulting in the accumulation of lamotrigine (Figure 7E). According to Drugbank, lamotrigine accumulates in the kidneys of male rats and may behave in a similar way in humans. This outcome may heighten the risk of drug toxicity and the occurrence of adverse events. The accumulation of lamotrigine resulting from decreased kidney function may be due to reduced excretion of the drug in the body, suggesting that dose adjustment may be required in the renal impairment population. In adults with renal impairment, the attainment of exposure levels equivalent to those observed in healthy individuals can be achieved through the administration of an appropriately adjusted dose of lamotrigine (Figure 7F). In our exploratory study, although the simulation deviates from the observed value by less than twofold, the PK profiles in different stages of kidney damage do not match well with the observed PK behavior. This discrepancy may be attributed to the varying pathological stages of kidney damage, which could impact the normal functioning of other organs, including hepatic elimination, thereby influencing lamotrigine metabolism. Currently, there is a paucity of real-world studies on lamotrigine in patients with renal impairment, underscoring the need for additional clinical data to verify our hypothesis. Therefore, based on our results, it is necessary to consider the PK behavior in patients with renal function and adjust the dosage according to the model prediction, validating the model in a real-world clinical setting to prove the reliability of the model [48].

Although the new PBPK model possesses distinctive advantages, it is imperative to recognize its inherent limitations. Additional research is necessary to improve the PBPK model. First, the model’s applicability is restricted to children aged two years and above due to insufficient knowledge regarding UGT’s role in lamotrigine metabolism in neonates and infants. Future studies may facilitate the extension of these models to neonates and infants. Second, there are limited observational data available to validate the reliability of the child model. Consequently, it is imperative to gather relevant data in the future to validate and optimize PBPK models for improving clinical medication decision-making recommendations. Thirdly, UGT2B10, a major UGT2B isoform, is the second major UGT enzyme involved in the N2-glucuronidation of lamotrigine in a novel study. The potential impact of UGT2B10 in healthy and special people given lamotrigine exposure will also be interrogated in a follow-up study shortly. Ultimately, the impact of kidney transporters in the model was not taken into consideration due to their age-dependent expression and the limited availability of information on patients with kidney damage, potentially compromising the accuracy of the predictions. The absence of precise clinical data on PK behavior in patients with varying degrees of renal impairment necessitates further validation of established models’ recommended dosages through well-designed clinical trials. Such studies can provide guidance on the administration of lamotrigine in patients with renal damage and potentially address gaps in clinical data to some extent.

## 4. Materials and Methods

### 4.1. Software and Data

The PBPK model was developed using PK-Sim^®^ modeling software (version 11.0, part of the OSP Suite, Leverkusen, Germany; https://www.open-systems-pharmacology.org/, accessed on 20 March 2025). Four key types of data were obtained from the literature and Drugbank, namely, drug-specific properties, virtual human populations, trial designs, and plasma concentration–time (*C*–*T*) curves after oral administration. The digitization of the observed drug plasma concentration–time data from scientific references was performed using GetData Graph Digitizer version 2.26 (getdata-graph-digitizer.com, accessed on 7 February 2025). The processing of data and the creation of graphics were executed utilizing Origin version 2018 (OriginLab, Northampton, MA, USA) software.

### 4.2. Establishment of PBPK Model in Adults After Oral Administration of Lamotrigine

This study employed a guidance-based workflow to develop a PBPK model for lamotrigine in healthy adults following oral administration (Tables S7 and S8). The model development process involved meticulous selection and integration of specific parameters, encompassing the physicochemical and absorption–distribution–metabolism-excretion (ADME) properties of lamotrigine. The essential physicochemical parameters and significant in vitro data utilized in the development of the PBPK model for lamotrigine were curated from the referenced literature and documented in a comprehensive compound file. Further elaboration on the origin and magnitude of these parameters can be found in Table S9 [49]. Cellular permeability was determined utilizing the standard PK-Sim^®^ algorithm, while tissue-to-plasma partition coefficients were determined employing the Rodgers and Rowland method [50]. Drug metabolism was integrated into the model by incorporating Michaelis–Menten parameter values, specifically the Michaelis constant (*K*_m_) and the maximum rate of reaction (*V*_max_), for all pertinent enzymes. In the simulations for model development, the characteristics of the virtual subjects (individuals and population) bore a resemblance to the demographic characteristics of participants in real clinical trials, encompassing age ranges, gender proportions, and dosing regimens. The age-dependent anatomical and physiological parameters of humans used in the simulation were incorporated into the PK-sim database, such as organ volumes matched to age and body weight and blood perfusion rates. The empirical data for the oral dosing model were derived from a previously published PK study [24], in which subjects were administered a single 100 mg oral dose of lamotrigine in solution form. Subsequently, a comparison was conducted between the predicted values of the models and the observed values from relevant studies. Following the successful recovery of observed data in various populations as documented in the existing literature, additional exploratory simulations were conducted to evaluate the suitability and therapeutic exposure of dosing regimens recommended by the WHO and the US FDA for adults (Table S10).

### 4.3. Development and Validation of PBPK Models for Pediatric Population Oral Lamotrigine Pharmacokinetics

Once the adult lamotrigine PBPK model was verified, trial simulations were conducted for the pediatric population to assess the properties of the PBPK model after a single oral dose of 2 mg/kg of lamotrigine. Firstly, drug-specific characteristics were maintained unaltered in children and adults when extrapolating the model to pediatrics. Age-specific physiological parameters of children older than 2 years of age (including organ size, blood flow, and tissue composition) were selected in the base model database of PK-Sim^®^. The model was extrapolated to pediatric patients aged 2 to 12 years old. The population characteristics and dosing regimens consistent with previous clinical trials were incorporated into PK-sim. The simulations were conducted in the pediatric population to assess the properties of the PBPK model after a single oral dose of 2 mg/kg of lamotrigine with a sample size of 100 individuals [37]. The default parameters pertaining to physiology and anatomy in PK-sim were derived from the International Commission on Radiological Protection (ICRP) population data to create virtual pediatric cohorts. The performance of the PBPK model was assessed by comparing the simulated *C*–*T* profiles from observed data from clinical research involving children aged 2 years and older, employing the same verification methodology as for the adult model. Additional exploratory simulations were performed on the established pediatric model to investigate drug exposure in children aged 2 years and older under long-term, multi-dose administration, utilizing the FDA’s recommended dosages (Table S10).

### 4.4. PBPK Model Scaling for the Renal Impairment Population

The PBPK model for lamotrigine was subjected to a further comparison using previous clinical data from patients with renal dysfunction. The estimation of kidney function, which is a crucial factor in the diagnosis of chronic kidney disease (CKD), is based on the glomerular filtration rate (GFR) of serum creatinine. The physiological parameters and characteristics of patients with kidney injury required to establish a kidney injury model of lamotrigine are described in the supplementary information (Table S11) [48,51,52]. The model was established based on a real-world PK study reported by Wootton et al. to predict lamotrigine PK in patients with different levels of kidney damage [38]. PBPK models were developed in adults with varying degrees of kidney insufficiency following a single dose of 200 mg. The vital PK parameters were predicted and compared with those of the normal population using box–whisker analysis. Doses for renal injury populations were adjusted based on the results of dose normalization for healthy adults to achieve identical exposure levels. Recommendations for dosing regimens were provided for patients with varying degrees of renal impairment to ensure adequate exposure and efficacy.

## 5. Conclusions

In this study, adult PBPK models for lamotrigine were successfully developed based on observed PK characteristics from the literature. Caution should be exercised when increasing the dose during initial and long-term therapy in patients with epilepsy, especially children, to prevent the risk of excessive blood concentrations and to minimize adverse events. The developed PBPK model was used to derive total plasma exposures and concentration–time profiles and provide dosing recommendations for renally impaired individuals. Our study presents a valuable model strategy for clinicians assessing the PK alterations of lamotrigine and facilitating TDM to enhance clinical efficacy while minimizing therapeutic risk. In conclusion, the established PBPK models combined actual conditions, such as physiological characteristics and pathological parameters of clinical patients. These models may serve as tools to assist clinicians in implementing personalized TDM, providing strategies for the clinical treatment of epilepsy and coping with the challenges of related factors.

## Data Availability

The original contributions presented in this study are included in the article/Supplementary Material. Further inquiries can be directed to the corresponding authors.

## Supplementary Materials

The following supporting information can be downloaded at https://www.mdpi.com/article/10.3390/ph18050637/s1: Figure S1: Simulations of pharmacokinetics of lamotrigine at a series of doses in healthy humans after a single oral administration. Prediction of plasma concentration-time profiles in pediatric patients with a dosage of 100 (A), 100 (B), 30 (C), 60 (D), 120 (E), 240 (F) mg compared with the observed data from reference. Figure S2: The predictions of lamotrigine mean plasma concentration-time profiles in healthy humans after multiple oral administrations compared with the observed data from reference. Figure S3: The sensitivity analysis of model parameters to the AUC (A) and Cmax (B) of lamotrigine. Figure S4: The simulation of plasma concentration profiles in adult and children (2 to 12 years of age) according to escalation regimen of FDA for lamotrigine.; Table S1: Comparison between observed and predicted pharmacokinetic parameters of lamotrigine with single dosage in adults. Table S2: Summary of lamotrigine pharmacokinetic parameters in adult with multiple dosage of lamotrigine in clinical studies and comparison with model predicted values. Table S3: Clinical studies used in building and evaluation of the pediatric PBPK models with single dosage of lamotrigine. Table S4: Observed and simulated Pharmacokinetic parameters of lamotrigine in children. Table S5: Clinical studies used to develop and evaluate the adult PBPK model with different renal functions. Table S6: Observed and simulated pharmacokinetic parameters of lamotrigine after oral administration in adult population with different renal functions. Table S7: The clinical pharmacokinetic studies used to develop and evaluate the adult PBPK model with single dosage of lamotrigine. Table S8: Overview of clinical studies used for building and evaluation of the adult PBPK models with multiple dosage of lamotrigine. Table S9: Summary of physicochemical parameters of lamotrigine used to establish the PBPK model. Table S10: Escalation Regimen of FDA for lamotrigine in adult and children (2 to 12 years of age). Table S11: The change of the physiological parameters and characteristics in the chronic kidney disease module.

## References

[1] Karoly P.J., Rao V.R., Gregg N.M., Worrell G.A., Bernard C., Cook M.J., Baud M.O. (2021). Cycles in epilepsy. Nat. Rev. Neurol..

[2] Lybrand Z.R., Goswami S., Zhu J., Jarzabek V., Merlock N., Aktar M., Smith C., Zhang L., Varma P., Cho K.-O. (2021). A critical period of neuronal activity results in aberrant neurogenesis rewiring hippocampal circuitry in a mouse model of epilepsy. Nat. Commun..

[3] Couzin-Frankel J. (2019). Foiling epilepsy in a brain at risk. Science.

[4] Devinsky O., Jones N.A., Cunningham M.O., Jayasekera B.A.P., Devore S., Whalley B.J. (2024). Cannabinoid treatments in epilepsy and seizure disorders. Physiol. Rev..

[5] Schevon C., Michalak A. (2023). Demystifying interictal discharges and seizure initiation in focal epilepsy. Brain.

[6] Hullett P.W., Lowenstein D.H. (2024). Major advances in epilepsy research in 2023. Lancet Neurol..

[7] Johannesen K.M. (2023). From precision diagnosis to precision treatment in epilepsy. Nat. Rev. Neurol..

[8] Husein N., Thijs R.D., Bunschoten J.W., Keezer M.R., Sander J.W. (2021). Concerns about lamotrigine. Lancet Neurol..

[9] Li R., Zhou Q., Ou S., Wang Y., Li Y., Xia L., Pan S. (2020). Comparison of long-term efficacy, tolerability, and safety of oxcarbazepine, lamotrigine, and levetiracetam in patients with newly diagnosed focal epilepsy: An observational study in the real world. Epilepsy Res..

[10] Mitra-Ghosh T., Callisto S.P., Lamba J.K., Remmel R.P., Birnbaum A.K., Barbarino J.M., Klein T.E., Altman R.B. (2020). PharmGKB summary: Lamotrigine pathway, pharmacokinetics and pharmacodynamics. Pharmacogenet. Genom..

[11] Chen J., Huang L., Zeng L., Jiang Z., Xiong M., Jia Z.-J., Cheng G., Miao L., Zhao L., Zhang L. (2024). The reference range of lamotrigine in the treatment of epilepsy in children: A systematic review. Eur. J. Clin. Pharmacol..

[12] Naik G.S., Kodagali R., Mathew B.S., Thomas M., Prabha R., Mathew V., Fleming D.H. (2015). Therapeutic drug monitoring of levetiracetam and lamotrigine: Is there a need?. Ther. Drug Monit..

[13] Pirie D.A., Al Wattar B.H., Pirie A.M., Houston V., Siddiqua A., Doug M., Bagary M., Greenhill L., Khan K.S., McCorry D. (2014). Effects of monitoring strategies on seizures in pregnant women on lamotrigine: A meta-analysis. Eur. J. Obstet. Gynecol. Reprod. Biol..

[14] Small B.G., Johnson T.N., Rowland Yeo K. (2023). Another step toward qualification of pediatric physiologically based pharmacokinetic models to facilitate inclusivity and diversity in pediatric clinical studies. Clin. Pharmacol. Ther..

[15] Heimbach T., Chen Y., Chen J., Dixit V., Parrott N., Peters S.A., Poggesi I., Sharma P., Snoeys J., Shebley M. (2021). Physiologically-based pharmacokinetic modeling in renal and hepatic impairment populations: A pharmaceutical industry perspective. Clin. Pharmacol. Ther..

[16] Verscheijden L.F.M., Koenderink J.B., Johnson T.N., de Wildt S.N., Russel F.G.M. (2020). Physiologically-based pharmacokinetic models for children: Starting to reach maturation?. Pharmacol. Ther..

[17] Dallmann A., Pfister M., van den Anker J., Eissing T. (2018). Physiologically based pharmacokinetic modeling in pregnancy: A systematic review of published models. Clin. Pharmacol. Ther..

[18] Luzon E., Blake K., Cole S., Nordmark A., Versantvoort C., Berglund E.G. (2017). Physiologically based pharmacokinetic modeling in regulatory decision-making at the European Medicines Agency. Clin. Pharmacol. Ther..

[19] Caleffi-Marchesini E.R., Borghi-Pangoni F.B., Macente J., Chiamulera-Mantovani P., Mazucheli J., Cristofoletti R., Diniz A. (2023). Exploring in vitro solubility of lamotrigine in physiologically mimetic conditions to prospect the in vivo dissolution in pediatric population. Biopharm. Drug Dispos..

[20] Caleffi-Marchesini E.R., Herling A.A., Macente J., Bonan R.H., Lima P.d.F., Moreno R., Alexandre V., Charbe N.B., Borghi-Pangoni F.B., Cristofoletti R. (2024). Adult and pediatric physiologically-based biopharmaceutics modeling to explain lamotrigine immediate release absorption process. CPT Pharmacomet. Syst. Pharmacol..

[21] Yeung C.H.T., Ito S., Autmizguine J., Edginton A.N. (2021). Incorporating breastfeeding-related variability with physiologically based pharmacokinetic modeling to predict infant exposure to maternal medication through breast milk: A workflow applied to lamotrigine. AAPS J..

[22] Birnbaum A.K., Kriel R.L., Im Y., Remmel R.P. (2001). Relative bioavailability of lamotrigine chewable dispersible tablets administered rectally. Pharmacotherapy.

[23] van Luin M., Colbers A., Verwey-van Wissen C.P., van Ewijk-Beneken-Kolmer E.W., van der Kolk M., Hoitsma A., da Silva H.G., Burger D.M. (2009). The effect of raltegravir on the glucuronidation of lamotrigine. J. Clin. Pharmacol..

[24] Incecayir T., Agabeyoglu I., Gucuyener K. (2007). Comparison of plasma and saliva concentrations of lamotrigine in healthy volunteers. Arzneimittelforschung.

[25] Srichaiya A., Longchoopol C., Oo-Puthinan S., Sayasathid J., Sripalakit P., Viyoch J. (2008). Bioequivalence of generic lamotrigine 100-mg tablets in healthy Thai male volunteers: A randomized, single-dose, two-period, two-sequence crossover study. Clin Ther..

[26] Ebert U., Thong N.Q., Oertel R., Kirch W. (2000). Effects of rifampicin and cimetidine on pharmacokinetics and pharmacodynamics of lamotrigine in healthy subjects. Eur. J. Clin. Pharmacol..

[27] Cohen A.F., Land G.S., Breimer D.D., Yuen W.C., Winton C., Peck A.W. (1987). Lamotrigine, a new anticonvulsant: Pharmacokinetics in normal humans. Clin. Pharmacol. Ther..

[28] Hamilton M.J., Cohen A.F., Yuen A.W., Harkin N., Land G., Weatherley B.C., Peck A.W. (1993). Carbamazepine and lamotrigine in healthy volunteers: Relevance to early tolerance and clinical trial dosage. Epilepsia.

[29] Jann M.W., Hon Y.Y., Shamsi S.A., Zheng J., Awad E.A., Spratlin V. (2006). Lack of pharmacokinetic interaction between lamotrigine and olanzapine in healthy volunteers. Pharmacotherapy.

[30] Van Der Lee M.J., Dawood L., Ter Hofstede H.J., Degraaffteulen M., Vanewijkbenekenkolmer E., Caliskanyassen N., Koopmans P., Burger D. (2006). Lopinavir/ritonavir reduces lamotrigine plasma concentrations in healthy subjects. Clin. Pharmacol. Ther..

[31] Almeida L., Nunes T., Sicard E., Rocha J.-F., Falcão A., Brunet J.-S., Lefebvre M., Soares-Da-Silva P. (2010). Pharmacokinetic interaction study between eslicarbazepine acetate and lamotrigine in healthy subjects. Acta Neurol. Scand..

[32] Li Y., Zhang F., Xu Y., Hu J., Li H. (2018). Pharmacokinetics, safety, and tolerability of lamotrigine chewable/dispersible tablet following repeat-dose administration in healthy chinese volunteers. Clin. Pharmacol. Drug Dev..

[33] Chien S., Yao C., Mertens A., Verhaeghe T., Solanki B., Doose D.R., Novak G., Bialer M. (2007). An interaction study between the new antiepileptic and CNS drug carisbamate (RWJ-333369) and lamotrigine and valproic acid. Epilepsia.

[34] Sidhu J., Job S., Bullman J., Francis E., Abbott R., Ascher J., Theis J.G. (2006). Pharmacokinetics and tolerability of lamotrigine and olanzapine coadministered to healthy subjects. Br. J. Clin. Pharmacol..

[35] Colucci R., Glue P., Holt B., Banfield C., Reidenberg P., Meehan J.W., Pai S., Nomeir A., Lim J., Lin C.C. (1996). Effect of felbamate on the pharmacokinetics of lamotrigine. J. Clin. Pharmacol..

[36] Gastrup S., Stage T.B., Fruekilde P.B., Damkier P. (2016). Paracetamol decreases steady-state exposure to lamotrigine by induction of glucuronidation in healthy subjects. Br. J. Clin. Pharmacol..

[37] Chen C., Casale E.J., Duncan B., Culverhouse E.H., Gilman J. (1999). Pharmacokinetics of lamotrigine in children in the absence of other antiepileptic drugs. Pharmacotherapy.

[38] Wootton R., Soul-Lawton J., Rolan P.E., Sheung C.T., Cooper J.D., Posner J. (1997). Comparison of the pharmacokinetics of lamotrigine in patients with chronic renal failure and healthy volunteers. Br. J. Clin. Pharmacol..

[39] Johnson T.N., Small B.G., Rowland Yeo K. (2022). Increasing application of pediatric physiologically based pharmacokinetic models across academic and industry organizations. CPT Pharmacomet. Syst. Pharmacol..

[40] Meesters K., Balbas-Martinez V., Allegaert K., Downes K.J., Michelet R. (2024). Personalized dosing of medicines for children: A primer on pediatric pharmacometrics for clinicians. Paediatr. Drugs.

[41] Salerno S.N., Capparelli E.V., McIlleron H., Gerhart J.G., Dumond J.B., Kashuba A.D.M., Denti P., Gonzalez D. (2023). Leveraging physiologically based pharmacokinetic modeling to optimize dosing for lopinavir/ritonavir with rifampin in pediatric patients. Pharmacotherapy.

[42] Batchelor H., European Paediatric Formulation Initiative (EUPFI) (2014). Paediatric biopharmaceutics classification system: Current status and future decisions. Int. J. Pharm..

[43] Patsalos P.N., Spencer E.P., Berry D.J. (2018). Therapeutic drug monitoring of antiepileptic drugs in epilepsy: A 2018 update. Ther. Drug Monit..

[44] Patsalos P.N., Berry D.J., Bourgeois B.F., Cloyd J.C., Glauser T.A., Johannessen S.I., Leppik I.E., Tomson T., Perucca E. (2008). Antiepileptic drugs—Best practice guidelines for therapeutic drug monitoring: A position paper by the subcommission on therapeutic drug monitoring, ILAE commission on therapeutic strategies. Epilepsia.

[45] Bohnert A.S.B., Walton M.A., Cunningham R.M., Ilgen M.A., Barry K., Chermack S.T., Blow F.C. (2018). Overdose and adverse drug event experiences among adult patients in the emergency department. Addict. Behav..

[46] Ni J., Tang X., Chen L. (2023). Medication overdose data analysis: A review of medication error reports in the FDA adverse event reporting system (FAERS). BMC Pharmacol. Toxicol..

[47] Zamir A., Alqahtani F., Rasool M.F. (2024). Chronic kidney disease and physiologically based pharmacokinetic modeling: A critical review of existing models. Expert Opin. Drug Metab. Toxicol..

[48] Franchetti Y., Nolin T.D. (2020). Dose optimization in kidney disease: Opportunities for PBPK modeling and mimulation. J. Clin. Pharmacol..

[49] Argikar U.A., Remmel R.P. (2009). Variation in glucuronidation of lamotrigine in human liver microsomes. Xenobiotica.

[50] Rodgers T., Leahy D., Rowland M. (2005). Physiologically based pharmacokinetic modeling 1: Predicting the tissue distribution of moderate-to-strong bases. J. Pharm. Sci..

[51] Rowland Yeo K., Aarabi M., Jamei M., Rostami-Hodjegan A. (2011). Modeling and predicting drug pharmacokinetics in patients with renal impairment. Expert. Rev. Clin. Pharmacol..

[52] Malik P.R.V., Yeung C.H.T., Ismaeil S., Advani U., Djie S., Edginton A.N. (2020). A physiological approach to pharmacokinetics in chronic kidney disease. J. Clin. Pharmacol..

