# Supplementary material for: Physiologically Based Pharmacokinetic Modeling to Predict Lamotrigine Exposure in Special Populations to Facilitate Therapeutic Drug Monitoring and Guide Dosing Regimens
Source: Pharmaceuticals (Basel). 2025 Apr 27;18(5):637. doi: 10.3390/ph18050637 (PMC12114674; doi:10.3390/ph18050637)
Supplement: Supplementary file 1 [file pharmaceuticals-18-00637-s001.zip › pharmaceuticals-3581110-supplementary.pdf]

Electronic Supplementary Information

Physiologically-based pharmacokinetic modeling to predict lamotrigine exposure in special populations to facilitate therapeutic drug monitoring and guide dosing regimen

Ji-Cheng Li, Chen-Fang Miao, Yun Lei*, Ai-Lin Liu*

Department of Pharmaceutical Analysis, Higher Educational Key Laboratory for Nano Biomedical Technology of Fujian Province, The School of Pharmacy, Fujian Medical University, Fuzhou, 350122, China

*Corresponding author: E-mail: leiyun@fjmu.edu.cn; ailinliu@fjmu.edu.cn.

S1. Methods

1.1. Validation of PBPK models for adult oral lamotrigine pharmacokinetics

The virtual clinical trial was performed to validate the performance of PBPK model in PK-Sim to promote confidence in the model parametrization. The included population characteristics (e.g., age, body weight), dosages, and dosing regimens mimicked those described in the observational studies (Table S7 and S8). The predicted concentration-time curves were visually compared with the observed data from a clinical study involving adults who received single and multiple-doses at different dose levels for initial verification [1–17]. Subsequently, the efficacy of PBPK models was assessed by employing the 2-fold error range criterion to compare clinically observed PK parameters, including the AUC, $C_{\max}$, $T_{\max}$ and $T_{1/2}$, with their model-predicted counterparts. To quantify the degree of error, the fold error (the ratio of observed to model-predicted values) was computed for each parameter by using the equation given below. The model was deemed successful if it satisfied the widely accepted 0.5- to 2.0-fold threshold. Such a criterion is generally considered appropriate for PK predictions and also commonly reported by other investigators.

$$\text{Fold error /ratio} = \frac{\text{Predicted value o f PK parameter}}{\text{Observed value o f PK parameter}}$$

1.2. Sensitivity analysis

By simulating the pharmacokinetic characteristics of lamotrigine 100 mg orally administered to health conditions, the sensitivity of model parameters to Lamotrigine AUC and $C_{\max}$ was predicted and analyzed. For reasons of numerical stability, a sensitivity is calculated as the average of several sensitivities based on different variations, the relative variations are defined by multiplication of the value in the simulation with variation factors (1.10, 1.05, 1/1.05, and 1/1.10). The sensitivity for the PK Parameter to that input parameter is then calculated as the ratio of the relative

change of that PK Parameter and the relative variation of the input parameter. With a 10% change in the parameter being evaluated, the relative change in AUC or C_{max} is reported as a sensitivity coefficient: for example, a sensitivity of -1.0 means that a 10% increase in the parameter results in a 10% decrease in the AUC or C_{max} value, and a sensitivity of + 0.5 means that a 10% increase in the parameter results in a 5% increase in AUC or C_{max}

1.3. PBPK model scaling to renal impairment population

The definition of the normal kidney function in a healthy individual is a GFR greater than 90 mL/min per 1.73 m^2. The PK-sim software was utilized to establish criteria for renal impairment, including moderate kidney injury (GFR ranging from 30 to less than 60 mL/min per 1.73 m^2), severe kidney injury (GFR ranging from 15 to less than 30 mL/min per 1.73 m^2), and end-stage renal disorder (GFR less than 15 mL/min per 1.73 m^2). The physiological parameters and characteristics, including GFR, kidney volume, kidney blood flow, hematocrit, gastric emptying time, and small intestinal transit time was displayed in the chronic kidney disease module of PK-Sim (Table S11) [18–20]. The changes of glomerular filtration fraction (f_{GFR}) and creatinine clearance rate (CL_{cr}), renal clearance in populations with renal impairment the characteristics of the clinical data for European patients with inadequate renal function were obtained using the formula.

$$f_{GFR} = \frac{CL_{\mathrm{R}}}{f_u \times \mathrm{GFR}}$$

$$CL_{R,i} = CL_{R,i} \times \frac{CLcr,i}{CLcr,j}$$

where CL_{R} represents the value of observed renal clearance, f_u represents the value of fraction unbound, and GFR represents the value of glomerular filtration rate.

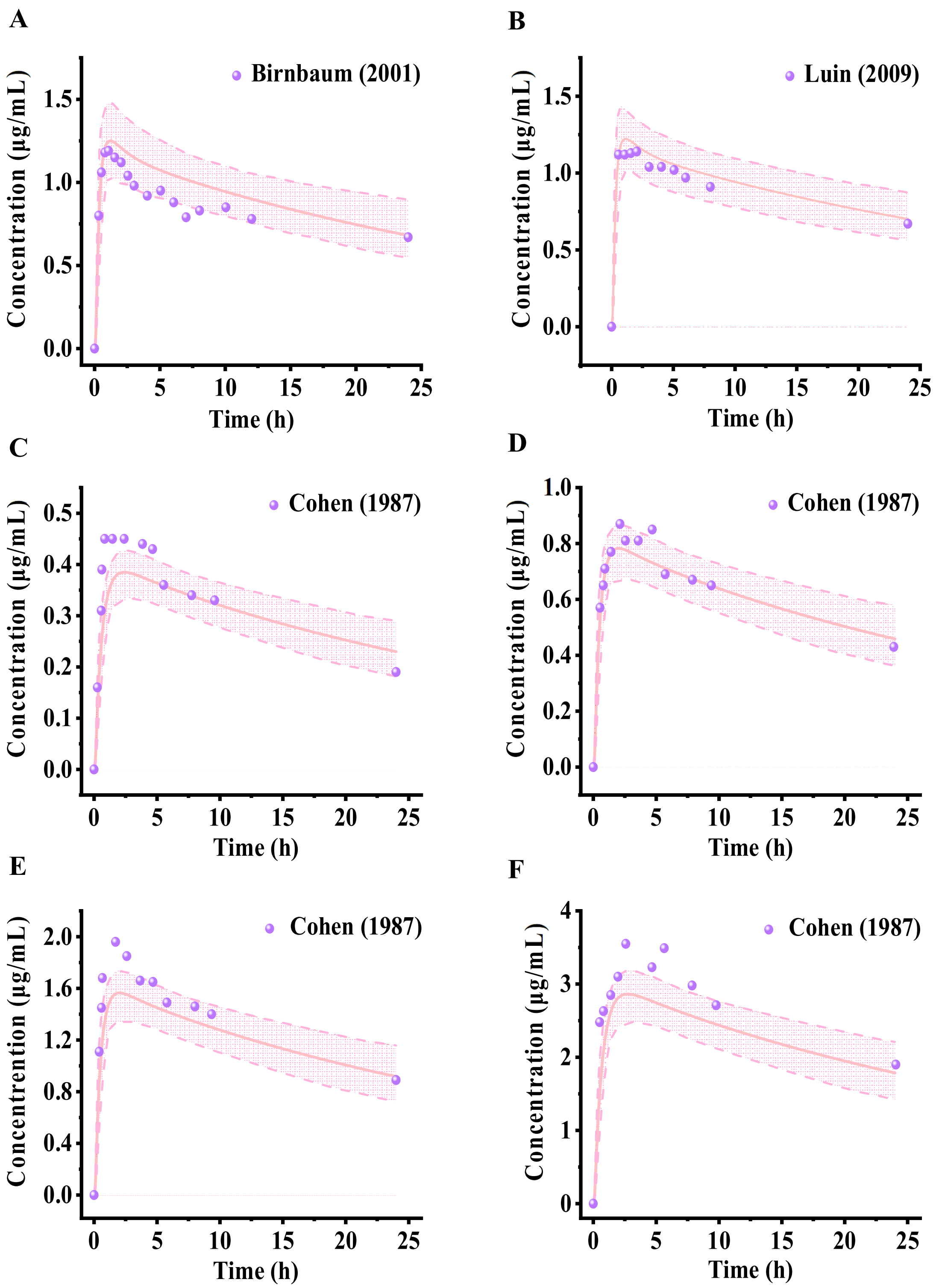

Figure S1. Simulations of pharmacokinetics of lamotrigine at a series of doses in healthy humans after a single oral administration. Prediction of plasma concentration-time profiles in pediatric patients with a dosage of 100 (**A**, [1]), 100 (**B**, [2]), 30 (**C**, [7]), 60 (**D**, [7]), 120 (**E**, [7]), 240 (**F**, [7]) mg compared with the observed data from reference. The observed data are from the published clinical study and are shown as

solid purple circles. The pink line represents the simulated mean plasma concentration-time profile. The shaded area of light pink represents the 5th and 95th confidence interval of the simulated mean concentrations.

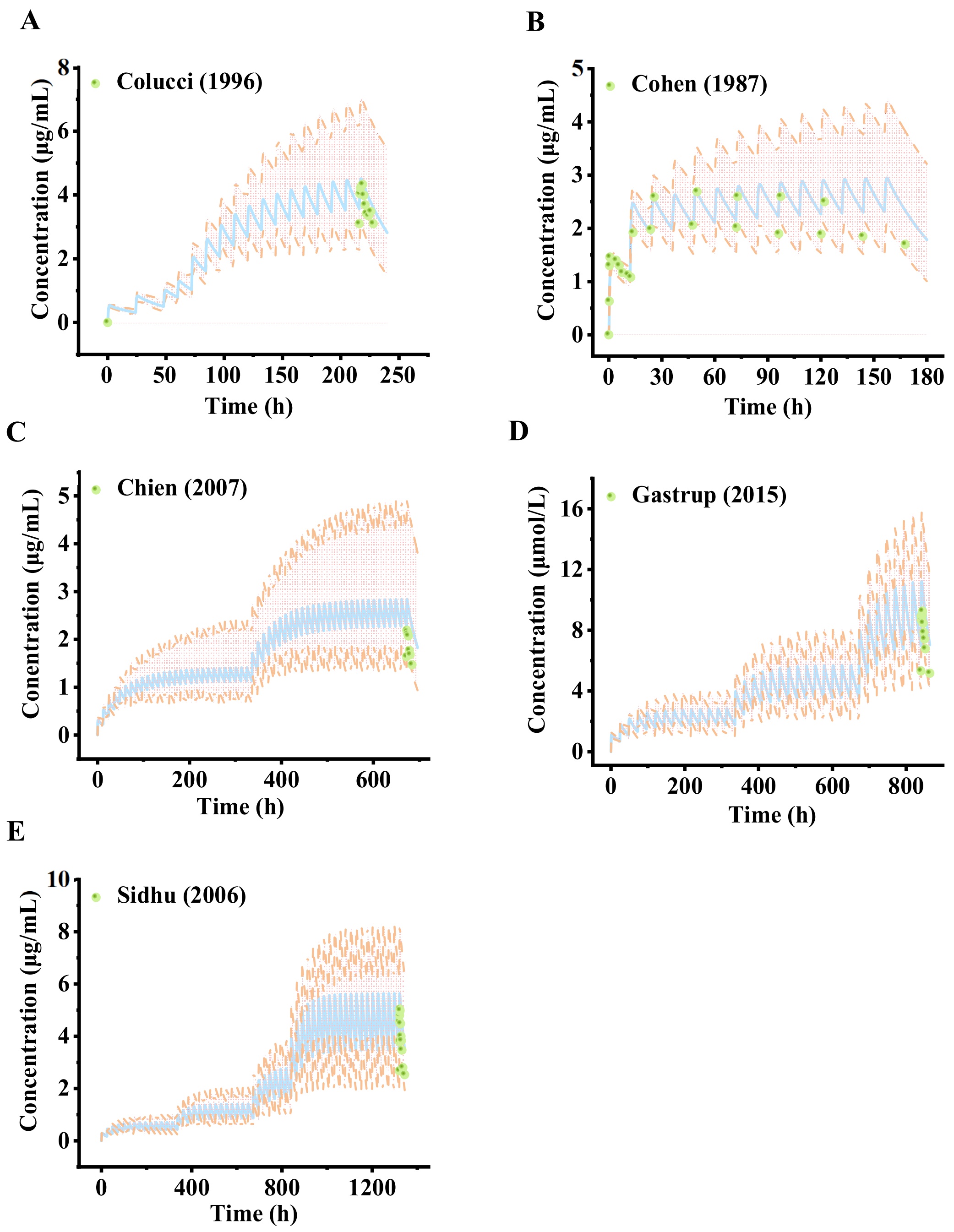

Figure S2. The predictions of lamotrigine mean plasma concentration-time profiles in healthy humans after multiple oral administrations compared with the observed data from reference. (**A**, [7]; **B**, [9]; **C**, [10]; **D**, [13]; **E**, [14]) The observed data are from the published clinical study and indicated as solid green circles. The light blue line represents the simulated mean plasma concentration-time profile. The area between the two orange dotted lines represents the 5th and 95th confidence interval of the simulated mean concentrations.

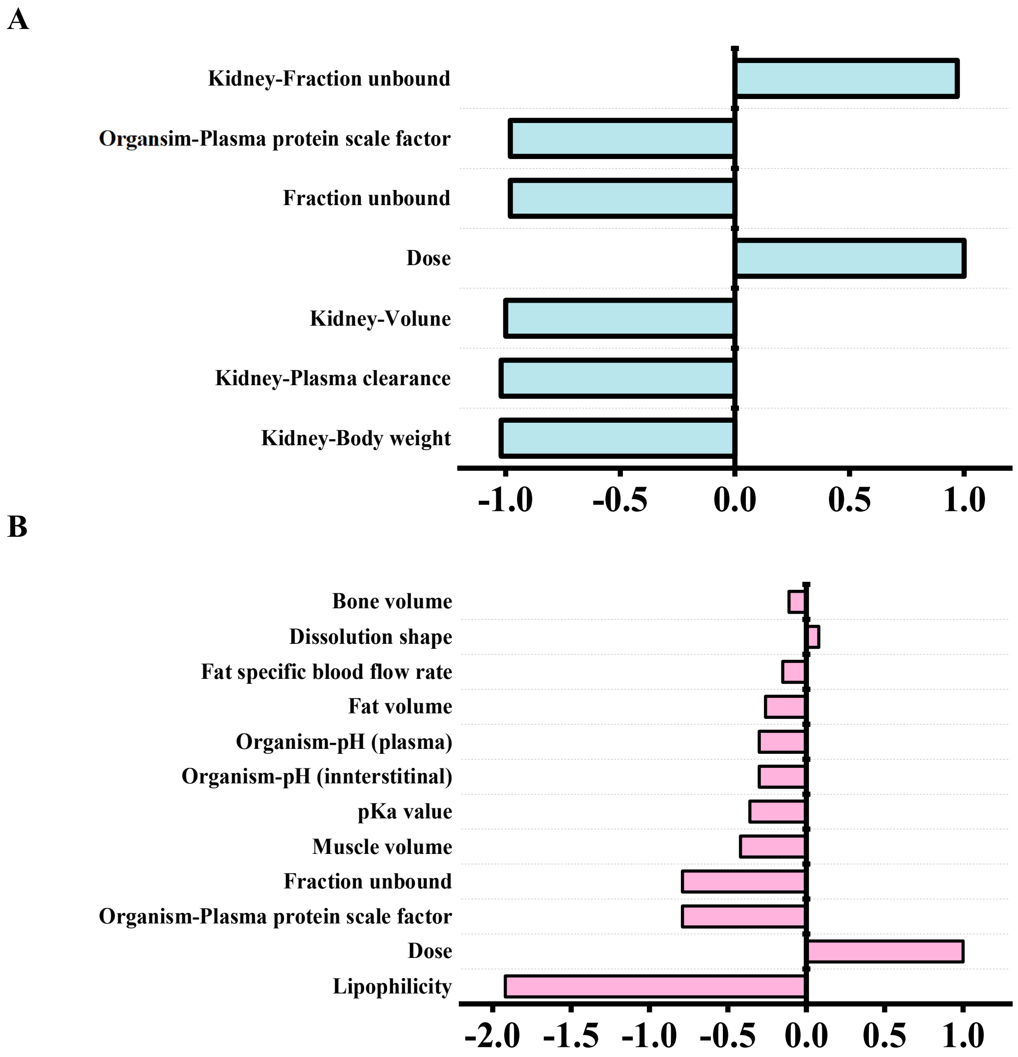

Figure S3. The sensitivity analysis of model parameters to the AUC (A) and C_{max} (B) of lamotrigine.

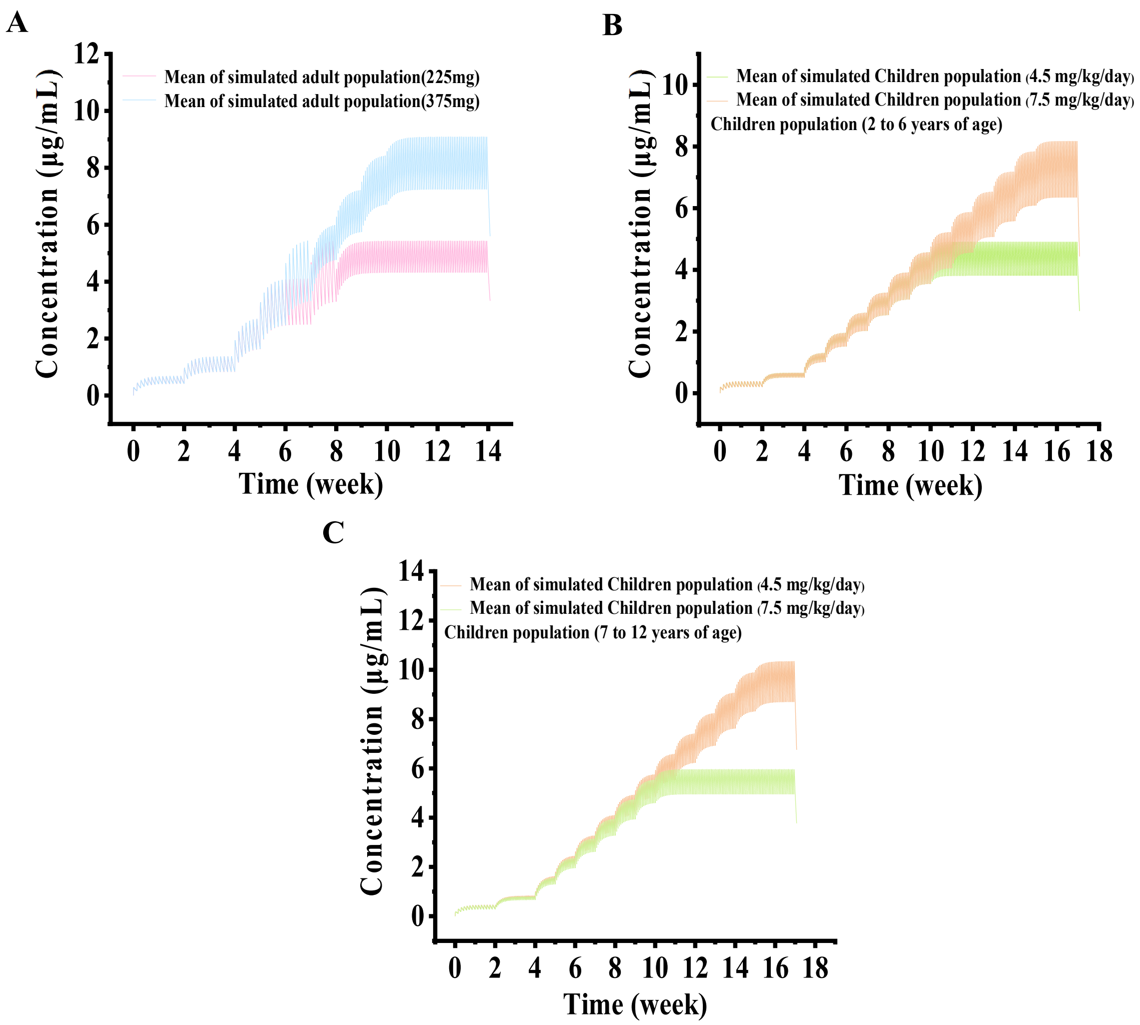

Figure S4. The simulation of plasma concentration profiles in adult and children (2 to 12 years of age) according to escalation regimen of FDA for lamotrigine.

Table S1 Comparison between observed and predicted pharmacokinetic parameters of lamotrigine with single dosage in adults

AUC (μg·h/mL)			C_{max} (μg/mL)			Half-life (h)			T_{max} (h)			Reference
Observed	Predicted	Fold-error	Observed	Predicted	Fold-error	Observed	Predicted	Fold-error	Observed	Predicted	Fold-error	
54.94	51.49	1.07	1.43	1.25	1.14	-	30.25	-	0.79	1.20	0.66	[1]
33.10	21.79	1.51	1.28	1.22	1.05	35.00	33.40	1.05	-	1.15	-	[2]
123.0	163.04	0.75	2.91	2.66	1.09	32.30	42.56	0.76	2.4	1.95	1.23	[3]
74.30	73.81	1.00	1.70	1.57	1.09	34.70	33.68	1.03	1.4	1.25	1.12	[4]
11.73	13.83	0.85	0.29	0.28	1.04	23.80	34.62	0.69	1.60	1.90	0.84	[5]
-	156.65	-	3.4	3.35	1.01	32.00	32.75	0.98	2.2	1.95	1.12	[6]
16.32	17.12	0.95	0.40	0.38	1.05	28.80	29.89	0.96	1.9	2.5	0.76	[7]
33.96	34.24	0.99	0.80	0.78	1.02	29.30	29.90	0.98	1.7	2.0	0.85	
66.00	68.49	0.96	1.60	1.56	1.02	29.10	29.90	0.97	2.1	2	1.05	
152.2	136.65	1.11	3.16	2.86	1.10	35.00	31.84	1.10	3.10	2.75	1.13	

AUC, area under the concentration-time curve; C_{max}, maximum concentration.

Table S2 Summary of lamotrigine pharmacokinetic parameters in adult with multiple dosage of lamotrigine in clinical studies and comparison with model predicted values

AUC (μg·h/mL)			C_{max} (μg/mL)			Half-life (h)			T_{max} (h)			Reference
Observed	Predicted	Fold-error	Observed	Predicted	Fold-error	Observed	Predicted	Fold-error	Observed	Predicted	Fold-error	
73.72	81.84	0.90	4.90	4.35	1.13	22.60	32.06	0.7	1.20	1.30	0.92	[8]
21.90	28.08	0.78	2.28	2.59	0.88	-	35.96	-	1.20	1.85	0.65	[9]
87.20	108.22	0.81	5.36	5.64	0.95	-	32.31	-	2.0	2.25	0.89	[10]
22.80	27.99	0.81	1.56	1.44	1.08	-	36.38	-	1.80	1.90	0.95	[11]
38.20	40.67	0.94	3.80	3.87	0.98	20.10	22.69	0.89	-	2.50	-	[12]
44.20	48.32	0.91	4.70	4.51	1.04	-	31.69	-	-	1.90	-	[13]
44.20	59.75	0.74	-	2.95	-	25.50	28.80	0.89	-	1.80	-	[7]
42.50	55.15	0.77	2.59	2.88	0.90	-	33.63	-	1.80	2.25	0.80	[14]
65.40	74.77	0.87	3.76	3.83	0.98	37.20	37.84	0.98	2.60	1.90	1.36	[15]

AUC, area under the concentration-time curve; C_{max}, maximum concentration.

Table S3 Clinical studies used in building and evaluation of the pediatric PBPK models with single dosage of lamotrigine

Number of participants (% female)	Age, years [mean/(range)]	Dose	Height, cm [mean/(rang)]	Weight, kg [mean/(rang)]	BMI, kg/m^2	Reference
-	7.6 (3.8-11.3)	2 mg/kg	124.0 (95.0-155.0)	27.9 (12.8-51.3)	-	[16]

Table S4 Observed and simulated Pharmacokinetic parameters of lamotrigine in children

Age range (year)	AUC (μg·h/mL)			C_{max} (μg/mL)			Half-life (h)			T_{max} (h)		
	Observed	Predicted	Fold-error	Observed	Predicted	Fold-error	Observed	Predicted	Fold-error	Observed	Predicted	Fold-error
3.8-11.3	61.00	57.21	1.07	1.48	1.34	1.10	32.30	28.13	1.15	4.10	3.00	1.37
<6	42.20	45.67	0.92	1.10	1.28	0.86	30.50	22.25	1.36	4.50	4.00	1.13
>6	70.40	63.39	1.11	1.60	1.56	1.03	33.20	25.64	1.29	3.90	4.00	0.98

AUC, area under the concentration-time curve; C_{max}, maximum concentration.

Table S5 Clinical studies used to develop and evaluate the adult PBPK model with different renal functions

Number of participants (% female)	Age, years [mean/(range)]	Dose	Height, cm [mean/(rang)]	Weight, kg [mean/(rang)]	BMI, kg/m^2	Reference
-	36-58	200 mg	-	-	-	[17]

Table S6 Observed and simulated pharmacokinetic parameters of lamotrigine after oral administration in adult population with different renal functions

Physiological status	AUC (μg·h/mL)			C_{max} (μg/mL)			Half-life (h)			T_{max} (h)		
	Observed	Predicted	Fold-error	Observed	Predicted	Fold-error	Observed	Predicted	Fold-error	Observed	Predicted	Fold-error
Healthy	94.60	98.91	0.96	2.46	2.28	1.08	27.80	29.96	0.92	2.20	2.25	0.98
Moderate RI	103.10	125.67	0.82	2.51	2.24	1.12	35.90	39.13	0.92	2.1	2.25	0.93
Severe RI	103.10	149.51	0.69	2.51	2.05	1.22	35.90	50.49	0.71	2.1	2.50	0.84
ESRD	103.10	160.00	0.64	2.51	1.66	1.51	35.90	68.15	0.53	2.1	2.75	0.76

AUC, area under the concentration-time curve; C_{max}, maximum concentration; RI, renal impairment; ESRD, end-stage renal disease.

Table S7 The clinical pharmacokinetic studies used to develop and evaluate the adult PBPK model with single dosage of lamotrigine

Number of participants (% female)	Age, years [mean/(range)]	Dose	Height, cm [mean/(rang)]	Weight, kg [mean/(rang)]	BMI, kg/m^2	Reference
12 (8%)	(25-39.2)	100 mg	-	-	-	[1]
-	34 (20-52)	100 mg	-	79 (63.0-94.0)	24 (20-28)	[2]
14 (36%）	23 (20-28)	200 mg	-	-	-	[3]
24 (0%)	20.5 (19-24）	100 mg	172.8 (165.6-179.4)	62.5 (55.1-69.9)	20.9 (18-25)	[4]
-	25 (21-29)	25 mg	(170.0-189.0)	63.0-100.0	-	[5]
-	(21-37)	300 mg	-	-	-	[6]
-	36 (27-49)	30, 60, 120, 240 mg	-	84.9 (64.8-103.3)	-	[7]

Table S8 Overview of clinical studies used for building and evaluation of the adult PBPK models with multiple dosage of lamotrigine

Number of participants (% female)	Age, years [mean/(range)]	Dose	Height, cm [mean/(rang)]	Weight, kg [mean/(rang)]	BMI, kg/m^2	Reference
32 (0%)	36 (23-45)	50 mg (D1-D2)<br>150 mg (D3-D27)	173.6 (162-184)	78.5 (59.5-100.4)	25 (19-29)	[8]
24 (33%)	35 (20-60)	25 mg q12 h (D1-D14)<br>50 mg q12h (D15-D27.5)	180.3 (161.0-196.0)	76.8 (55.7-92.6)	23.6 (19-28)	[9]
52 (0%)	(18-42)	25 mg (D1-D14)<br>50 mg (D15-D28)<br>100 mg (D29-D35)<br>200 mg (D36-D56)	-	-	-	[10]
14 (14.30%)	24 (21.6-26.4)	25 mg (D1-D5)<br>50 mg (D6-D15)	-	78.9 (67.8-90.0)	-	[11]
24 (50%)	36.4 (19.3-	50 mg (D1-D2)	175 (157-200)	71 (53-88)	23 (18-29)	[12]

	63.6)	100 mg BID (D3-D10)				
-	24.5 (20-37)	50 mg (D1-D2)<br>100 mg BID (D3-D9)<br>100 mg (D10)	73.6 (62-88)	-	-	[13]
-	28 (19-37)	120 mg BID (D1)<br>60 mg BID (D2-D7)	-	72.6 (63.6-90.0)	-	[7]
-	25 (22-32)	25 mg (D1-D14)<br>50 mg (D15-D28)<br>100 mg (D29-D36)	-	-	22 (20-28)	[14]
16 (37.5%)	25.7 (18-45)	25 mg (D1-D14)<br>50 mg (D15-D28)<br>100 mg (D29-D42)	-	-	21.6 (19-24)	[15]

Table S9 Summary of physicochemical parameters of lamotrigine used to establish the PBPK model

**Parameter**	**Value**	**Source**
Molecular formula	$C_9H_7Cl_2N_5$	Drugbank[a]
Molecular weight (g/mol)	256.091	Drugbank[a]
logP[b]	1.87	Drugbank[a]
pKa[c]	5.5; 5.7	Drugbank[a]
Solubility (mg/mL)	0.17	Drugbank[a]
pH for solubility	7	Drugbank[a]
Fraction unbound	0.45	Drugbank[a]
Metabolic clearance parameters		
UGT1A3 V_{max} (pmol/min/mg of microsomal protein)	17	[21]
UGT1A3 K_m (μM)	70	
UGT1A4 V_{max} (pmol/min/mg of microsomal protein)	153	
UGT1A4 K_m (μM)	550	

[a] https://go.drugbank.com/drugs/DB0008

[b] logP: oil–water partition coefficients.

[c] pKa: acid dissociation constant.

Table S10 Escalation Regimen of FDA for lamotrigine in adult and children (2 to 12 years of age)

	Adult	Children (2 to 12 years of
Weeks 1 and 2	25 mg every day	0.3 mg/kg/day<br>in 1 or 2 divided doses
Weeks 3 and 4	50 mg/day	0.6 mg/kg/day<br>in 2 divided doses
Weeks 5 onwards to maintenance	Increase by 50 mg/day<br>every 1 to 2 weeks	The dose should be increased every 1 to<br>2 weeks as follows:<br>calculate 0.6 mg/kg/day
Usual Maintenance Dose	225 to 375 mg/day<br>(in 2 divided doses)	4.5 to 7.5 mg/kg/day<br>(Maximum 300 mg/day<br>in 2 divided doses)

Table S11 The change of the physiological parameters and characteristics in the chronic kidney disease module

Physiological parameters	The chronic kidney disease module			
	Normal kidney function	Moderate kidney injury	Severe kidney injury	End-stage renal disorder
Hematocrit	0.47	0.45	0.43	0.35
Plasma protein scale factor	1	1.07	1.16	1.55
Gastric emptying time (min)	15	15	24	24
Small intestinal transit time (h)	2.10	2.10	2.94	2.94
Kidney				
Specific blood flow rate (mL/min/100 g organ)	259.93	123.49	88.32	69.42
Blood flow rate (L/min)	1.21	0.41	0.23	0.15
Organ volumes (L)	0.46	0.33	0.26	0.22

References

1. Birnbaum AK, Kriel RL, Im Y, Remmel RP. Relative bioavailability of lamotrigine chewable dispersible tablets administered rectally. *Pharmacotherapy.* 2001;21:158-162. doi:10.1592/phco.21.2.158.34104
2. Van Luin M, Colbers A, Verwey-van Wissen CP, et al. The effect of raltegravir on the glucuronidation of lamotrigine. *J Clin Pharmacol.* 2009;49:1220-1227. doi:10.1177/0091270009345689
3. Incecayir T, Agabeyoglu I, Gucuyener K. Comparison of plasma and saliva concentrations of lamotrigine in healthy volunteers. *Arzneimittelforschung.* 2007;57:517-521. doi:10.1055/s-0031-1296641
4. Srichaiya A, Longchoopol C, Oo-Puthinan S, Sayasathid J, Sripalakit P, Viyoch J. Bioequivalence of generic lamotrigine 100-mg tablets in healthy Thai male volunteers: a randomized, single-dose, two-period, two-sequence crossover study. *Clin Ther.* 2008;30:1844-1851. doi:10.1016/j.clinthera.2008.10.018
5. Ebert U, Thong NQ, Oertel R, Kirch W. Effects of rifampicin and cimetidine on pharmacokinetics and pharmacodynamics of lamotrigine in healthy subjects. *Eur J Clin Pharmacol.* 2000;56:299-304. doi:10.1007/s002280000146
6. Hamilton MJ, Cohen AF, Yuen AW, et al. Carbamazepine and lamotrigine in healthy volunteers: relevance to early tolerance and clinical trial dosage. *Epilepsia.* 1993;34:166-173. doi:10.1111/j.1528-1157.1993.tb02393.x
7. Cohen AF, Land GS, Breimer DD, Yuen WC, Winton C, Peck AW. Lamotrigine, a new anticonvulsant: pharmacokinetics in normal humans. *Clin Pharmacol Ther.* 1987;42:535-541. doi:10.1038/clpt.1987.193
8. Almeida L, Nunes T, Sicard E, et al. Pharmacokinetic interaction study between eslicarbazepine acetate and lamotrigine in healthy subjects. *Acta Neurol Scand.* 2010;121:257-264. doi:10.1111/j.1600-0404.2009.01233.x
9. Chien S, Yao C, Mertens A, et al. An interaction study between the new antiepileptic and CNS drug carisbamate (RWJ-333369) and lamotrigine and valproic acid. *Epilepsia.* 2007;48:1328-1338. doi:10.1111/j.1528-1167.2007.01037.x
10. Sidhu J, Job S, Bullman J, et al. Pharmacokinetics and tolerability of lamotrigine and olanzapine coadministered to healthy subjects. *Br J Clin Pharmacol.* 2006;61:420-426. doi:10.1111/j.1365-2125.2006.02598.x
11. Jann MW, Hon YY, Shamsi SA, Zheng J, Awad EA, Spratlin V. Lack of pharmacokinetic interaction between lamotrigine and olanzapine in healthy volunteers. *Pharmacotherapy.* 2006;26:627-633. doi:10.1592/phco.26.5.627
12. Van Der Lee MJ, Dawood L,Ter Hofstede HJ, et al. Lopinavir/ritonavir reduces lamotrigine plasma concentrations in healthy subjects. *Clin Pharmacol Ther.* 2006;80:159-168. doi:10.1016/j.clpt.2006.04.014
13. Colucci R, Glue P, Holt B, et al. Effect of felbamate on the pharmacokinetics of lamotrigine. *J Clin Pharmacol.* 1996;36:634-638. doi:10.1002/j.1552-4604.1996.tb04228.x
14. Gastrup S, Stage TB, Fruekilde PB, Damkier P. Paracetamol decreases steady-state exposure to lamotrigine by induction of glucuronidation in healthy subjects. *Br J Clin Pharmacol.* 2016;81:735-741. doi:10.1111/bcp.12840
15. Li Y, Zhang F, Xu Y, Hu J, Li H. Pharmacokinetics, safety, and tolerability of lamotrigine chewable/dispersible tablet following repeat-dose administration in healthy chinese volunteers. *Clin Pharmacol Drug Dev.* 2018;7:627-633. doi:10.1002/cpdd.449
16. Chen C, Casale EJ, Duncan B, Culverhouse EH, Gilman J. Pharmacokinetics of lamotrigine in children in the absence of other antiepileptic drugs. *Pharmacotherapy*. 1999;19:437-441. doi:10.1592/phco.19.6.437.31052
17. Wootton R, Soul-Lawton J, Rolan PE, Sheung CT, Cooper JD, Posner J. Comparison of the pharmacokinetics of lamotrigine in patients with chronic renal failure and healthy volunteers. *Br J Clin Pharmacol*. 1997;43:23-27. doi:10.1111/j.1365-2125.1997.tb00028.x
18. Zamir A, Alqahtani F, Rasool MF. Chronic kidney disease and physiologically based pharmacokinetic modeling: a critical review of existing models. *Expert Opin Drug Metab Toxicol*. 2024;20:95-105. doi:10.1080/17425255.2024.2311154
19. Rowland Yeo K, Aarabi M, Jamei M, Rostami-Hodjegan A. Modeling and predicting drug pharmacokinetics in patients with renal impairment. *Expert Rev Clin Pharmacol.* 2011;4(2):261-274. doi:10.1586/ecp.10.143
20. Malik PRV, Yeung CHT, Ismaeil S, Advani U, Djie S, Edginton AN. A physiological approach to pharmacokinetics in chronic kidney disease. *J Clin Pharmacol.* 2020;60 Suppl 1:S52-S62. doi:10.1002/jcph.1713
21. Argikar, U.A.; Remmel, R.P. Variation in glucuronidation of lamotrigine in human liver microsomes. Xenobiotica 2009, 39, 355–363. https://doi.org/10.1080/00498250902745082
